# Microbial gut diversity in four grasshopper species and its correlation with cellulose digestibility

**DOI:** 10.3389/fmicb.2022.1002532

**Published:** 2022-11-10

**Authors:** Yao Ling, Wen-Jing Li, Fei-Fei Li, Xiao-Bao Xue, Yuan-Yi Gao, Li Wang, Ke Liang, Xin-Jiang Li

**Affiliations:** The Key Laboratory of Zoological Systematics and Application, School of Life Sciences, Institute of Life Sciences and Green Development, Hebei University, Baoding, China

**Keywords:** grasshopper, gut microbiota, diversity, cellulose digestibility, 16S rDNA

## Abstract

Grasshoppers are common pests, and their intestinal microbes have coevolved with them. These microorganisms have varied community structures, and they participate in the nutritional absorption and metabolism of grasshoppers. Here, we describe the gut microbiota diversity of four species of grasshoppers, *Oxya chinensis*, *Pararcyptera microptera meridionalis*, *Gastrimargus marmoratus*, and *Calliptamus abbreviatus.* We constructed a 16S rDNA gene library and analyzed the digestibility of cellulose and hemicellulose in grasshoppers using moss black phenol and anthrone colorimetry. The grasshopper with the highest microbial diversity in the gut among the four species was *Oxya chinensis*, and there were no significant differences in gut microbial diversity between the two geographic collections of *Oxya chinensis*. The most dominant phyla of the four grasshopper gut microorganisms were Proteobacteria, Bacteroidetes, and Firmicutes, and the most dominant genus was *Enterobacter*. The gut microbiota features of the four grasshoppers were correlated with their cellulose and hemicellulose digestibility. There was a significant positive correlation with cellulose digestibility for *Pantoea*. A significant negative correlation was found with cellulose digestibility for *Acinetobacter*, *Enterococcus*, *Citrobacter*, *Serratia*. A significant negative correlation was found with hemicellulose digestibility for *Pantoea*. This study contributes to the understanding of the structural composition of different species of grasshoppers gut microbiota, which may be useful for developing grasshopper digestive tracts as bioreactors for cellulose decomposition, improving the decomposition and utilization of agricultural straw, producing clean biomass energy, and processing biologically derived products.

## Introduction

Insects are one of the most numerous, evolutionarily oldest, and widely distributed animal groups in nature. There are more than one million species of insects, making them the most diverse group of animals (Mei et al., 2018). Correspondingly, insects have evolved complex and highly specialized intestinal systems to digest a variety of food sources; thus, the insect gut system has been designated as the world’s smallest natural bioreactor (Breznak and Brune, 1994; Dar et al., 2021). The complex structure and large surface area to volume ratio of the insect gut provides a habitat for many intestinal microorganisms and guarantees a rich biodiversity (Brune and Dietrich, 2015). Initially, insect gut microorganisms were sporadically studied in *Bombyx mori*, *Dendrolimus*, and *Apriona germari* (Cai, 1990). In recent years, with the recent advancement in molecular biology technologies, significant progress has been made regarding the diversity and biological functions of insect gut microorganisms. A large number and diversity of gut microbes have been identified in common insect species. Such microorganisms include bacteria, fungi, protozoa, and archaea, with bacteria being the dominant group in most insect gut microbiomes (Schloss et al., 2006).

The insect gut serves as a link between the host and the external environment, and the presence of a large number of intestinal microbes in the gut has a significant impact on the development (Shin et al., 2011; Huang et al., 2021), reproduction (Arredondo-Santoyo et al., 2020), thermotolerance (Brumin et al., 2011), drug resistance (Daisley et al., 2018; Xia et al., 2018), behavior, and survival of the insect host (Shi et al., 2014; Tan et al., 2015; Lavy et al., 2022). In Beard et al. (1993) successfully suppressed the ability of a vector (stink bug) to transmit Chagas disease by introducing a virulence protein gene into the symbiotic bacterium of the insect, allowing the modified symbiotic bacterium to colonize the vector. *Acinetobacter*, *Wolbachia*, and *Staphylococcus* contribute to insecticide metabolism in the gut of *Nilaparvata lugens*, and this finding has implications for pest control (Tang et al., 2020). In addition, *Serratia* inhibits *Plasmodium* reproduction by regulating gene expression and activates the immune system of mosquitoes against *Plasmodium* infection (Bai et al., 2019). Humus-feeding carrion beetles can cooperate with their gut microbes to protect food from spoilage (Shukla et al., 2018). Acevedo et al. (2017) isolated *Klebsiella* from the oral secretions of *Spodoptera frugiperda*, which down-regulated peroxidase activity and up-regulated trypsin inhibitor activity in tomato plants, resulting in a reduction in the ability of tomato plants to defend themselves against pests. Similarly, Smith et al. (2017) identified enzymes involved in carbohydrate metabolism and pathogen defense by studying the hindgut of the orthopteran insect Mormon Cricket (*Anabrus simplex*). Furthermore, Marín-Miret et al. (2022) identified microbial candidate genes that may be involved in environmental detoxification of plants by functional prediction of gut microbes in *Brithys crini*. In addition, insect gut microorganisms show potential for application in insect resource utilization and industrial production (Hosokawa et al., 2007; Whitten et al., 2016).

With the increasing attention paid to environmental protection, ecology, and resource recycling, the utilization of cellulose has been studied in different research fields. Cellulose is a polymer composed of D-glucoside and β-1,4-glucosidic bonds, which are the most widely distributed and abundant carbon sources on Earth; however, the high degree of polymerization and complex crystalline structure of cellulose makes it difficult to use, and the utilization of cellulose by humans remains low. Although physical and chemical methods can be used to effectively hydrolyze complex cellulose polymers, physicochemical treatments often require harsh and extreme conditions (Singh et al., 2015; Ma et al., 2020). The use of microorganisms for cellulose degradation has the advantages of high efficiency, no harm to the environment, mild reaction conditions, and diverse application prospects (Yang et al., 2021). Microorganisms catalyze the degradation of cellulose by secreting cellulase into the extracellular space. The use of cellulase to convert cellulose into small-molecule organic matter is an effective measure to utilize cellulose-based substances (Zheng, 2016; Jin et al., 2022). Most insects depend on symbiosis with intestinal microorganisms, such as bacteria or protozoa, to acquire the ability to degrade cellulose (Xie et al., 2014). Symbiotic microorganisms contribute to nitrogen fixation and cycling, intermediate product metabolism, and cellulose degradation in termites (Li et al., 2017). Most termites achieve 60–90% effective digestion of lignocellulose, with gut bacteria making a significant contribution (Calusinska et al., 2020). Termites, grasshoppers, and long-horned beetles feed on cellulose and are a natural resource for cellulose digestion. They possess a variety of cellulose-degrading symbiotic bacteria, and their ability to degrade lignin model compounds is approximately 20–100%, which is 30–40% higher than that of large herbivores (Zhang et al., 2021). Binoy et al. (2022) proposed the use of herbivorous insect gut microbes to degrade lignocellulose in waste plants to produce biogas. In addition, Suárez-Moo et al. (2020) identified functional genes related to nitrogen fixation, uric acid metabolism, and plant cell wall degradation in a study of the gut microbes of the adult Dung Beetle, *Copris incertus* (belonging to the family Chrysomelidae). Su et al. (2014) used denaturing gradient gel electrophoresis (DGGE) to study 16 symbiont species of the grasshopper gut and found that cellulolytic enzymes and gut microbial communities may indicate the association of different grasshopper species with their feeding patterns. These findings show the potential of intestinal microorganisms for cellulose degradation, and the study of microorganisms related to cellulose degradation in the intestine can lay the foundation for the practical degradation and utilization of cellulose.

Grasshoppers (Orthoptera: Acridoidea) are common agricultural pests, and their gut microorganisms have co-evolved to form diverse population structures and perform biological functions (e.g., nutrient uptake and metabolism; Yun et al., 2014; Lavy et al., 2020). In this study, two geographic populations of *Oxya chinensis* (Thunberg, 1815) (b = Baoding, c = Chengde. OCb and OCc are abbreviations of *Oxya chinensis* collected in Baoding, Hebei and Chengde, Hebei, respectively), *Pararcyptera microptera meridionalis* (Ikonnikov, 1911) (PM), *Gastrimargus marmoratus* (Thunberg, 1815) (GM), and *Calliptamus abbreviatus* Ikonnikov, 1913 (CA) were used as experimental animals to study gut microbial diversity and to uncover the composition, structure, and functional status of such gut microorganisms. The determination and analysis of the cellulose digestibility of different grasshopper species and their relationship with intestinal microorganisms will allow us to study the genera of cellulose- and hemicellulose-degrading bacteria in the grasshopper intestine. These insights will elucidate grasshopper digestion and metabolism (useful for pest control) and facilitate the development of grasshopper intestines as bioreactors to produce biomass energy and bio-based products using cellulose-degrading bacteria.

## Materials and methods

### Intestinal microbial diversity of grasshoppers

#### Collection and processing of experimental materials

Adult grasshoppers were used in this experiment. The field collection sites of grasshoppers used in the experiments all belong to temperate continental monsoon climate. Specific specimen collection information is presented in Table 1.

**Table 1:** Experimental specimens.

Species	Sample code		Sequencing region	Collection date	Locality	Temperature (°C)
*Oxya chinensis* (Thunberg, 1815)	OCc	OC1	V1–V9	September, 2021	Chengde, China	27 ± 3
		OC2		September, 2021		
		OC3		September, 2021		
	OCb	OC4		August, 2021	Baoding, China	30 ± 3
		OC5		August, 2021		
		OC6		August, 2021		
*Pararcyptera microptera meridionalis* (Ikonnikov, 1911)	PM	PM1	V1–V9	July, 2021	Zhangjiakou, China	26 ± 4
		PM2		July, 2021		
		PM3		July, 2021		
*Gastrimargus marmoratus* (Thunberg, 1815)	GM	GM1	V1–V9	July, 2021	Baoding, China	32 ± 3
		GM2		July, 2021		
		GM3		July, 2021		
*Calliptamus abbreviatus* Ikonnikov, 1913	CA	CA1	V1–V9	July, 2021	Baoding, China	32 ± 3
		CA2		July, 2021		
		CA3		July, 2021		

OCb and OCc are abbreviations of *Oxya chinensis* collected in Baoding, Hebei and Chengde, Hebei, respectively, b = Baoding, c = Chengde.

Grasshoppers collected in the wild were housed in separate cages by species and starved for 1–2 days to empty the grasshoppers’ intestines of feces. The entire intestine of one female and two male grasshoppers was collected as one sample under aseptic conditions, as detailed by Wang et al. (2020). The grasshopper intestines collected in this experiment were grouped according to region and species, and a total of five sample groups were collected, including three biological replicates in one group of samples, which were labeled and stored at-80°C.

#### DNA extract

The total DNA of the intestinal contents was extracted with the TGuide S96 Magnetic Soil/Stool DNA Kit (Tiangen Biotech (Beijing) Co., Ltd., China) according to manufacturer instructions. The DNA concentration of the samples was measured with the Qubit dsDNA HS Assay Kit and Qubit 4.0 Fluorometer (Invitrogen, Thermo Fisher Scientific, Oregon, United States).

#### Construction of the 16S rDNA gene library

The 27F: AGRGTTTGATYNTGGCTCAG and 1492R: TASGGHTACCTTGTTASGACTT universal primer set was used to amplify the full-length 16S rRNA gene from the genomic DNA extracted from each sample (Guo et al., 2021). Both the forward and reverse 16S primers were tailed with sample-specific PacBio barcode sequences to allow for multiplexed sequencing. We chose to use barcoded primers (S1 in the Supplementary material) because this reduces chimera formation as compared to the alternative protocol in which primers are added in a second PCR reaction. The KOD One PCR Master Mix (TOYOBOLife Science) was used to perform 25 cycles of PCR amplification, with initial denaturation at 95°C for 2 min, followed by 25 cycles of denaturation at 98°C for 10 s, annealing at 55°C for 30 s, and extension at 72°C for 1 min 30 s, and a final step at 72°C for 2 min. The total of PCR amplicons were purified with Agencourt AMPure XP Beads (Beckman Coulter, Indianapolis, IN, United States) and quantified using the Qubit dsDNA HS Assay Kit and Qubit 4.0 Fluorometer (Invitrogen, Thermo Fisher Scientific, Oregon, United States). After the individual quantification step, amplicons were pooled in equal amounts. SMRTbell libraries were prepared from the amplified DNA by SMRTbell Express Template Prep Kit 2.0 according to the manufacturer’s instructions (Pacific Biosciences). Purified SMRTbell libraries from the pooled and barcoded samples were sequenced on a single PacBio Sequel II 8 M cell using the Sequel II Sequencing kit 2.0. This process was completed by the Beijing Biomarker Technologies Co., Ltd.

#### Bioinformatics analysis

The bioinformatics analysis of this study was performed with the aid of the BMK Cloud (Biomarker Technologies Co., Ltd., Beijing, China). The raw reads generated from sequencing were filtered and demultiplexed using the SMRT Link software (version 8.0) with the minPasses ≥ 5 and minPredictedAccuracy ≥ 0.9, in order to obtain the circular consensus sequencing (CCS) reads. Subsequently, the lima (version 1.7.0) was employed to assign the CCS sequences to the corresponding samples based on their barcodes. CCS reads containing no primers and those reads beyond the length range (1,200–1,650 bp) were discarded through the recognition of forward and reverse primers and quality filtering using the Cutadapt quality control process (version 2.7). The UCHIME algorithm (v8.1) was used in detecting and removing chimera sequences to obtain the clean reads (See the Data Availability Statement for the raw data and S2 in the supplementary material for the metadata file sample information). In order to keep all sample sequences at the same depth and eliminate the differences in species composition caused by different sequencing depths, the sequences were randomly selected from the samples according to the minimum number of sequences in all samples. The number of species represented by these sequences was counted, and the sample Rarefaction Curve was constructed based on the number of sequences and the number of species (S3 in the Supplementary material). Sequences with similarity ≥ 97% were clustered into the same operational taxonomic unit (OTU) by USEARCH (v10.0; Edgar, 2013), and the OTUs with reabundance < 0.005% were filtered (Bokulich et al., 2012) (S4 in the Supplementary material). Shannon index curve was generated by Mothur and R package (S5 in the Supplementary material). Taxonomy annotation of the OTUs was performed based on the Naive Bayes classifier in QIIME2 using the SILVA database (release 132) with a confidence threshold of 70%. The Alpha diversity were calculated and displayed by the QIIME2 and R software, respectively. Beta diversity was determined to evaluate the degree of similarity of microbial communities from different samples using QIIME. UPGMA, nonmetric multidimensional scaling (NMDS) and Adonis analysis were used to analyze the beta diversity. Furthermore, we employed Linear Discriminant Analysis (LDA) effect size (LEfSe) to test the significant taxonomic difference among group. The LEfSe analysis was performed using R and the Psych, Pheatmap and reshape2 package (Kostic et al., 2015) on the Biomarker Cloud Platform. LEfSe analysis generated LDA effective size (Default threshold: > 4) and *p*-value (Default threshold: less 0.05), and then generated corresponding pictures on the Biomarker Cloud Platform. PICRUSt2 was applied to perform species annotation on feature sequences based on reference phylogenetic tree. Potential functions and functional genes in samples were predicted based on Integrated Microbial Genomes (IMG) database. The significance of difference in function abundance between samples was evaluated by G-TEST (Number of annotated functional genes > 20) in STAMP. Threshold for significant difference was set as p-value smaller than 0.05.

### Cellulose and hemicellulose digestibility

Field-collected grasshoppers were grouped according to species, with *Oxya chinensis* collected from the two areas divided into two groups, for a total of five groups. Grasshoppers were fed fresh wheat seedlings (*Triticum aestivum* Linnaeus, 1753) grown in the laboratory, and the specific feeding procedure, preparation of fecal sample solution, and wheat seedling sample solution were performed as described by Wang et al. (2020). The dry-to-fresh ratio of the wheat seedlings was determined as stipulated by Subrata et al. (2020).

Glucose and xylose standard curves were plotted with reference (Wang et al., 2020). The linear equation of glucose obtained in this experiment was *y* = 1.7321*x* + 0.0083, *R*^2^ = 0.9955, and the linear equation for xylose was *y* = 53.96*x*–0.0044, *R*^2^ = 0.9969. “*x*” is the reducing sugar concentration and “*y*” is the absorbance value.

The absorbance values of the reaction solutions of fecal and wheat seedling samples were determined using the anthrone and moss black phenol colorimetric methods, respectively, as per Wang et al. (2020). The cellulose and hemicellulose digestibility of grasshoppers on wheat seedlings were calculated using the following equations:


$$\begin{array}{l} \mathrm{Cellulose}\;\left(\mathrm{hemicellulose}\right)\;\mathrm{content}\;\left(\%\right)\\ =\frac{c\times 240\times 10^{-3}L\times \mathrm{dilution mutiple}\times 0.9\left(0.88\right)}{m}\times 100\%\text{.} \end{array}$$

$$\mathrm{Cellulose}\;\left(\mathrm{hemicellulose}\right)\;\mathrm{digestibiliy}\;\left(\%\right)=\frac{a-b}{a}\times 100\%\text{.}$$

Note: *c* is the sugar concentration (g/l) calculated according to the standard curve, 240 is the total volume of sample solution (ml), *m* is the weighed sample mass (g). 0.9 and 0.88 are coefficients (Zhang et al., 2010), *a* is amount of cellulose (hemicellulose) fed on wheat seedlings (g), and *b* is fecal cellulose (hemicellulose) content (g).

### Gut microbes and digestibility correlation analysis

One of the purposes of this study is to explore the possibility that grasshopper gut can be used as a bioreactor for cellulose degradation through the correlation between gut microbes and cellulose digestibility, so as to provide ideas for further research on cellulose degrading bacteria. According to the analysis of sequencing results, the 20th most abundant genus only accounted for 2.58% of the intestinal microbial abundance in PM1, which were all 0 in the intestines of the remaining 17 samples (This data can be obtained in S8 of the Supplementary material). Therefore, to make the study more meaningful and representative, the relationship between grasshopper gut microorganisms and cellulose and hemicellulose digestibility was analyzed by subjecting the top 20 genera in relative abundance in the gut of the four grasshopper species to Spearman correlation analysis (*γ* = 0.1; *p* = 0.05) with cellulose and hemicellulose digestibility of grasshoppers, and correlation heat maps were constructed. The analysis was performed using the Biomarker Cloud Platform (Biomarker Biotechnology Co., Beijing, China).

## Result

### 16S rDNA sequencing analysis

**Figure 1:**
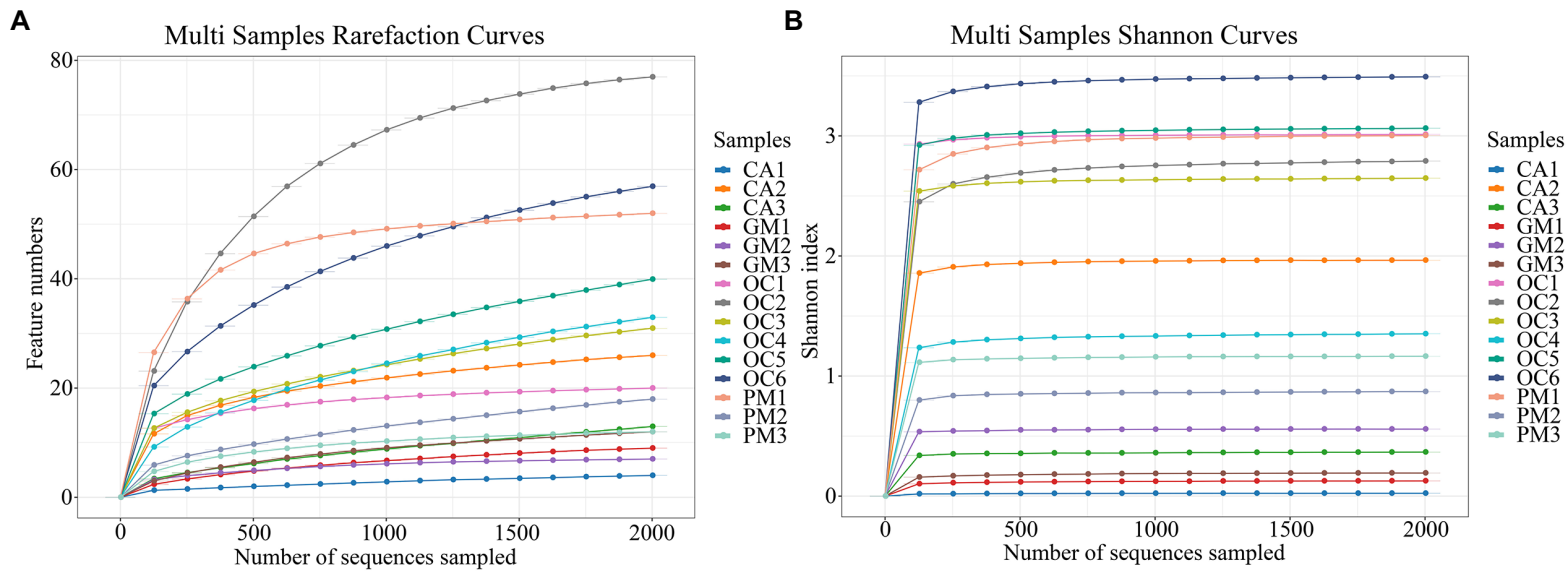
**(A)** Sample Rarefaction Curves. *X*-axis: Counts of randomly sampled sequences; Y-axis: Counts of features detected by giving sequences (operational taxonomic unit numbers). **(B)** The Shannon index curves. *X*-axis: Counts of randomly sampled sequences; *Y*-axis: Corresponding Shannon index. As the amount of sequencing increases more species are discovered until after species saturation, increasing the number of sampling bars does not reveal new features. CA, *Calliptamus abbreviatus*; GM, *Gastrimargus marmoratus*; OC1–OC3, *Oxya chinensis* in Chengde; OC4–OC6, *Oxya chinensis* in Baoding; PM, *Pararcyptera microptera meridionalis*.

A total of 105,390 CCS sequences were obtained following the sequencing of 15 samples, and each sample generated at least 4,939 CCS sequences, with an average of 7,026 CCS sequences (Table 2). The sample Rarefaction Curve (Figure 1A) and Shannon index curve (Figure 1B) indicated that the sequencing volume was sufficient, sequencing depth was saturated, and increasing the sample volume did not produce more OTUs. Conclusively, sequencing data are reliable and sufficient for diversity analysis.

**Table 2:** Sequence statistics for the PacBio runs.

Sample ID	Raw CCS	Clean CCS	Effective CCS	AvgLen (bp)	Effective (%)
CA1	7,509	7,509	7,509	1,465	100
CA2	7,494	7,494	7,450	1,465	99.41
CA3	7,524	7,524	7,511	1,464	99.83
GM1	7,498	7,498	7,493	1,465	99.93
GM2	7,507	7,507	7,485	1,462	99.71
GM3	6,114	6,114	6,112	1,463	99.97
OC1	7,526	7,526	7,227	1,463	96.03
OC2	4,939	4,939	4,897	1,456	99.15
OC3	7,548	7,547	7,283	1,459	96.49
OC4	5,532	5,531	5,466	1,461	98.81
OC5	7,490	7,490	7,187	1,460	95.95
OC6	7,582	7,582	7,314	1,457	96.47
PM1	6,111	6,110	6,065	1,461	99.25
PM2	7,503	7,503	7,403	1,465	98.67
PM3	7,513	7,513	7,375	1,466	98.16

Sample ID: Sample ID; Raw-CCS: Counts of identified circular consensus sequencing (CCS) reads in the sample; Clean CCS: Counts of clean CCS reads (post primer removal and length filtration); Effective-CCS: Counts of effective CCS reads after chimeric read removal; AvgLen(bp): average length reads in the sample; effective (%): percentage of effective CCS reads in raw reads. CA, *Calliptamus abbreviatus*; GM, *Gastrimargus marmoratus*; OC1–OC3, *Oxya chinensis* in Chengde; OC4–OC6, *Oxya chinensis* in Baoding; PM, *Pararcyptera microptera meridionalis*.

### Species annotation and taxonomic analysis

Based on 97% sequence similarity, 159 OTUs belonged to one kingdom, 15 phyla, 25 orders, 48 orders, 73 families, 106 genera, and 139 species (Table 3).

**Table 3:** Species statistics at all levels of the samples.

Species	Sample	Seqs_Num	OTU_Num	Kingdom	Phylum	Class	Order	Family	Genus	Species
CA	CA1	2013	4	1	2	2	3	4	4	4
	CA2	2013	26	1	5	7	12	17	22	26
	CA3	2013	13	1	4	6	8	10	11	12
GM	GM1	2013	9	1	4	5	6	6	9	9
	GM2	2013	7	1	4	5	6	6	7	7
	GM3	2013	12	1	4	5	7	7	11	11
OCc	OC1	2013	20	1	5	7	8	11	12	17
	OC2	2013	77	1	11	17	28	37	60	77
	OC3	2013	31	1	6	7	11	13	19	30
OCb	OC4	2013	33	1	5	7	13	17	23	33
	OC5	2013	40	1	9	12	18	20	24	38
	OC6	2013	57	1	8	10	17	27	37	55
PM	PM1	2013	52	1	10	15	23	27	35	40
	PM2	2013	18	1	5	7	9	10	14	18
	PM3	2013	12	1	4	5	5	7	9	12
	Total	30,195	159	1	15	25	48	73	106	139

CA, CA1–CA3, *Calliptamus abbreviatus*; GM, GM1–GM3, *Gastrimargus marmoratus*; OCc, OC1–OC3, *Oxya chinensis* in Chengde; OCb, OC4–OC6, *Oxya chinensis* in Baoding; PM, PM1–PM3, *Pararcyptera microptera meridionalis*. OTU_Num: Feature (OTU/ASV) numbers; Seqs_Num: Feature reads.

According to the number of species levels in Table 3, it can be preliminarily determined that the grasshopper gut microbial diversity, in descending order, was *Oxya chinensis* (OCb), *Oxya chinensis* (OCc), *Pararcyptera microptera meridionalis* (PM), *Calliptamus abbreviatus* (CA), and *Gastrimargus marmoratus* (GM).

**Figure 2:**
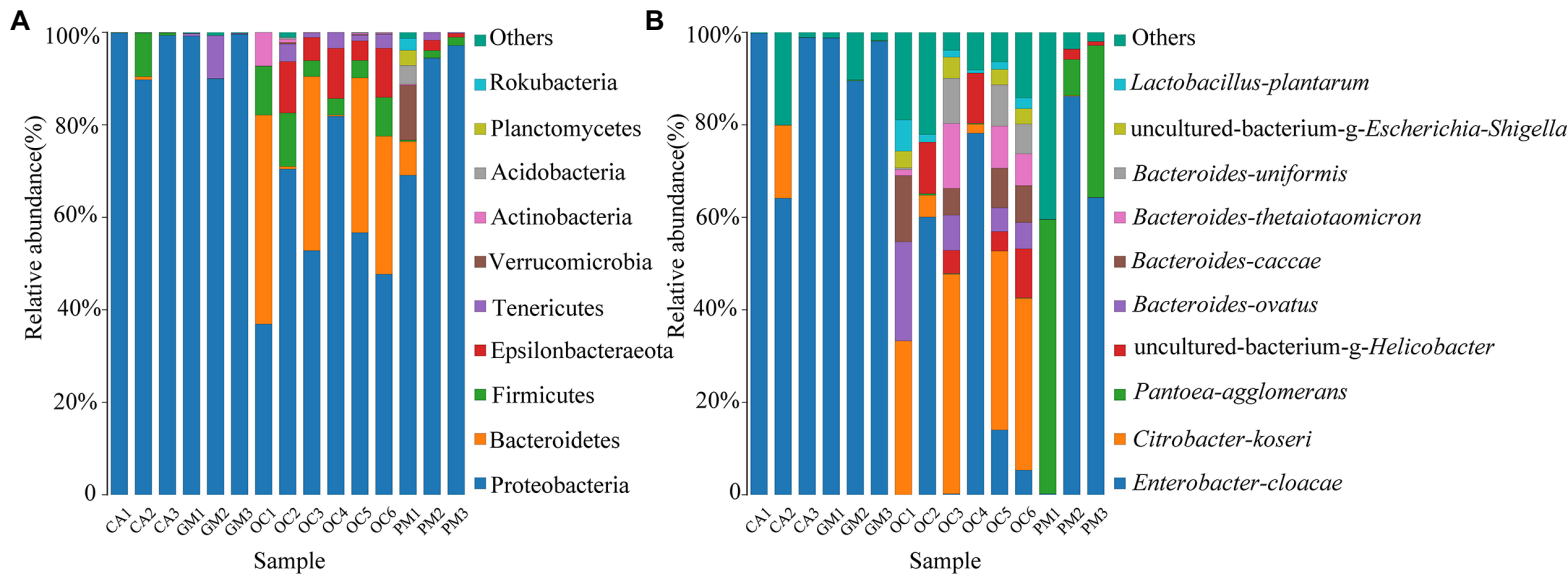
Bacterial composition (top 10 relative abundance values) at **(A)** the phylum level and **(B)** the species level. Each color represents a species, and the height of the color block indicates the proportion of the species in relative abundance. Other species are incorporated as “Others” shown in the diagram. CA, *Calliptamus abbreviatus*; GM, *Gastrimargus marmoratus*; OC1–OC3, *Oxya chinensis* in Chengde; OC4–OC6, *Oxya chinensis* in Baoding; PM, *Pararcyptera microptera meridionalis*.

Collectively the four grasshopper species had the following three dominant phyla: Proteobacteria, Bacteroidetes, and Firmicutes (Figure 2A). Proteobacteria accounted for the highest proportion of gut microorganisms in four species of grasshoppers, followed by *Bacteroidetes*, *Firmicutes*, *Epsilonbacteraeota*, *Tenericutes*, *Verrucomicrobia*, *Actinobacteria*, *Acidobacteria*, *Planctomycetes*, and *Rokubacteria*. There was no significant difference in the relative abundance of grasshopper gut microbiota at the phylum level (*p* > 0.1). The gut microorganisms of *Oxya chinensis* (OCc and OCb) from different regions did not differ significantly at the phylum level, but there were significantly more microorganisms in the gut of *Oxya chinensis* than in other grasshopper species, such as *Bacteroidetes* and *Epsilonbacteraeota*.

Figure 2B shows the top ten species with relative abundances at the species level. The gut of *Oxya chinensis* has the top ten species in relative abundance. The dominant species in the guts of the four grasshopper species differed, and the most common dominant species was *Enterobacter cloacae*. The largest proportion of *Enterobacter cloacae* was found in the guts of *Calliptamus abbreviatus* (CA) and *Gastrimargus marmoratus* (GM), while *Citrobacter koseri* was significantly more abundant in the guts of *Oxya chinensis* collected from different regions than other grasshopper species. The proportion of *Pantoea agglomerans* in the intestines of *Pararcyptera microptera meridionalis* (PM) was significantly higher than that of the other grasshopper species. This finding is consistent with the results of the previous analysis.

**Figure 3:**
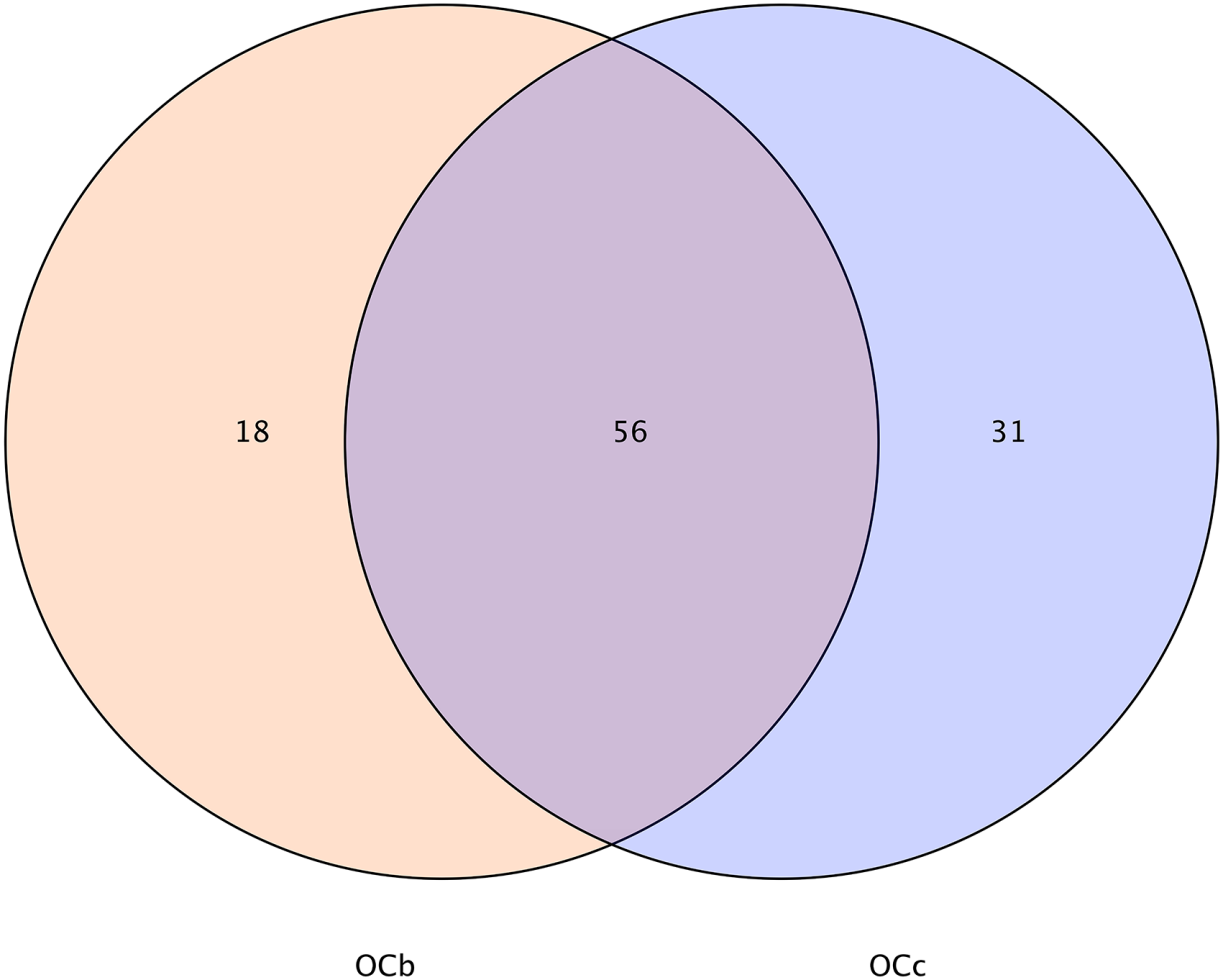
Characteristics of *Oxya chinensis* in Baoding and *Oxya chinensis* in Chengde described by a Venn diagram. The number of overlapping parts is the number of common features between the two groups of samples, and the number of non-overlapping parts is the number of unique features of the two groups of samples. OCb, *Oxya chinensis* in Baoding (b = Baoding); OCc, *Oxya chinensis* in Chengde (c = Chengde).

The gut microbes of *Oxya chinensis* (OCc and OCb) captured at the two sites in Chengde and Baoding were not significantly different at the phylum level (Figure 2A). To intuitively express the common and unique characteristics of the two gut microbes, we plotted Venn diagrams of *Oxya chinensis* (OCc and OCb) at the species level for both sites (Figure 3) (S6 in the Supplementary material). As shown in Figure 3, *Oxya chinensis* (OCc) and *Oxya chinensis* (OCb) shared 56 microbial species, and 31 endemic species in *Oxya chinensis* (OCc) were higher than 18 endemic species in *Oxya chinensis* (OCb). A one-way ANOVA on the number of microbial species in the gut of both sites revealed no significant differences (*p* > 0.05) in the gut species of *Oxya chinensis* (OCc and OCb) between the two sites.

### α/β-Diversity analysis

**Figure 4:**
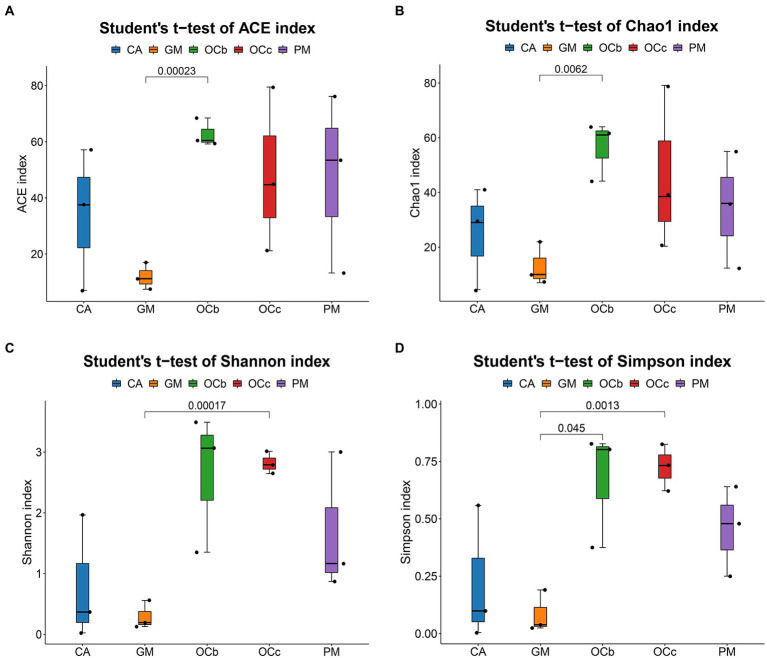
Boxplot of alpha diversity metrics of four species of grasshoppers. **(A)** ACE index of the four species grasshoppers. **(B)** Chao1 index of the four species grasshoppers. **(C)** Shannon index of the four species grasshoppers. **(D)** Simpson index of four species grasshoppers. In the box diagram, the meanings of each symbol are as follows: Upper and lower end lines of the box: Interquartile range (IQR); The median; Upper and lower edges: maximum and minimum inner circumference (1.5 times IQR); A point on the outside of the upper and lower edges: represents an outlier. The number on the line between the columns is the value of *p* of the *t*-test (if *p* > 0.05, the value of *p* will not be displayed by default). CA, *Calliptamus abbreviatus*; GM, *Gastrimargus marmoratus*; OCc, *Oxya chinensis* in Chengde (c = Chengde); OCb, *Oxya chinensis* in Baoding (b = Baoding); PM, *Pararcyptera microptera meridionalis*.

The coverage rates of the 15 grasshopper samples in the five groups were all above 99%, indicating that most species in the samples were identified (Table 4). Histograms of the differences between groups were plotted according to the α-diversity index (Table 4; Figures 4A–D). Figures 4A, B indicates that the number of microbial species in the grasshopper gut from high to low was: *Oxya chinensis* (OCb), *Oxya chinensis* (OCc), *Pararcyptera microptera meridionalis, Calliptamus abbreviatus*, and *Gastrimargus marmoratus*. The abundance of microbial in the gut of *Oxya chinensis* (OCb) was significantly higher than that of the *Gastrimargus marmoratus*. Higher values of Shannon’s and Simpson’s indices indicate a higher species diversity in the samples (Grice et al., 2009), and as can be seen Figures 4C,D, the grasshoppers with the highest to lowest microbial diversity in the grasshopper gut were, in descending order, *Oxya chinensis* (OCb), *Oxya chinensis* (OCc), *Pararcyptera microptera meridionalis, Calliptamus abbreviatus*, and *Gastrimargus marmoratus*. The intestinal microbial diversity of *Oxya chinensis* (OCc and OCb) was significantly higher than that of *Gastrimargus marmoratus*. Although the microbial diversity and abundance in the gut of *Oxya chinensis* collected from different regions differed, these differences were not significant. The diversity and abundance of microorganisms in the gut of *Oxya chinensis* (OCc and OCb) were the highest, followed by *Pararcyptera microptera meridionalis* (PM), *Calliptamus abbreviatus* (CA), and *Gastrimargus marmoratus* (GM). The α-diversity analysis was consistent with the results of the species annotation and taxonomic analysis.

**Table 4:** α-Diversity index statistics of grasshopper intestinal samples.

Sample ID	ACE	Chao1	Simpson	Shannon	Coverage
CA1	7.0000	4.5000	0.0040	0.0237	0.9990
CA2	37.4849	29.0000	0.5577	1.9650	0.9970
CA3	57.1387	41.0000	0.0990	0.3667	0.9960
GM1	11.2028	10.0000	0.0246	0.1269	0.9985
GM2	7.4444	7.0000	0.1900	0.5585	0.9995
GM3	16.9874	22.0000	0.0383	0.1924	0.9975
OC1	21.1541	20.3333	0.8244	3.0126	0.9990
OC2	79.4713	79.1538	0.6220	2.7910	0.9960
OC3	44.6915	38.5000	0.7321	2.6478	0.9950
OC4	60.4550	44.1429	0.3749	1.3518	0.9935
OC5	59.1983	61.0000	0.8016	3.0639	0.9925
OC6	68.4690	64.0000	0.8268	3.4922	0.9930
PM1	53.4192	55.0000	0.6402	3.0035	0.9980
PM2	76.1781	36.0000	0.2502	0.8707	0.9955
PM3	13.2448	12.3333	0.4788	1.1650	0.9990

CA, *Calliptamus abbreviatus*; GM, *Gastrimargus marmoratus*; OC1–OC3, *Oxya chinensis* in Chengde (OCc, c = Chengde); OC4–OC6, *Oxya chinensis* in Baoding (OCb, b = Baoding); PM, *Pararcyptera microptera meridionalis*.

**Figure 5:**
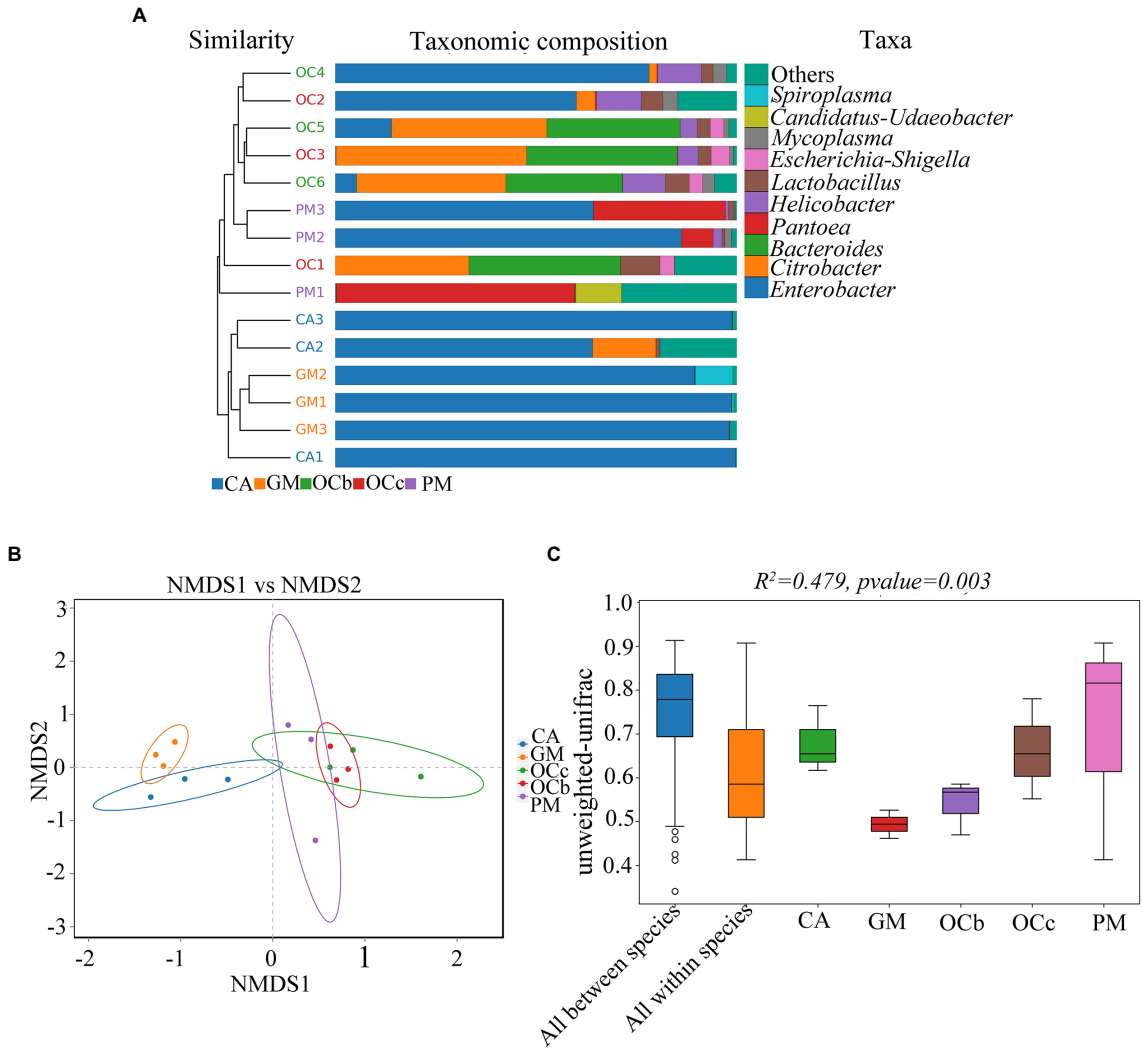
β-Diversity analysis. **(A)** Unweighted pair group method with arithmetic mean (UPGMA) cluster analysis of the four species grasshoppers. **(B)** Non-multidimensional scaling (NMDS) analysis. **(C)** Adonis analysis box plot. CA, *Calliptamus abbreviatus*; GM, *Gastrimargus marmoratus*; OCc, *Oxya chinensis* in Chengde (c = Chengde); OCb, *Oxya chinensis* in Baoding (b = Baoding); PM, *Pararcyptera microptera meridionalis*.

Figure 5A shows the combination of the UPGMA clustering tree and the abundance histogram at the genus level. From the clustering tree on the left, *Gastrimargus marmoratus* (GM) and *Calliptamus abbreviatus* (CA) were clustered into a group, and the biological replicates were also clustered, indicating that the gut microbial composition of the two grasshopper species was similar between individuals and groups. *Oxya chinensis* (OCb and OCc) and *Pararcyptera microptera meridionalis* (PM) clustered into a group, and the biological replicates clustered together except for OC1, indicating that the gut microbial compositions of the two grasshopper species, *Oxya chinensis* and *Pararcyptera microptera meridionalis* (PM), were similar. The top ten genera in relative abundance are shown on the right, with “others” representing the remaining genera. The dominant genus shared by the four grasshopper species in the gut is *Enterobacter*. The gut microbial diversity of *Oxya chinensis* (OCb and OCc) and *Pararcyptera microptera meridionalis* (PM) was significantly greater than that of *Gastrimargus marmoratus* (GM) and *Calliptamus abbreviatus* (CA). This was consistent with the results of the species annotation and taxonomic analysis. The proportion of *Citrobacter* and *Bacteroides* in the gut of *Oxya chinensis* was significantly higher than that of other grasshopper species, and *Pantoea* in the gut of *Pararcyptera microptera meridionalis* (PM) was significantly higher than that of other grasshopper species. The dominant genera in the gut of *Oxya chinensis* collected from different regions were similar, and among the top ten genera in relative abundance, *Oxya chinensis* had the most genera, followed by *Pararcyptera microptera meridionalis* (PM). Among the annotated intestinal microbiota, *Escherichia*–*Shigella* and *Bifidobacterium* are endemic to *Oxya*, and *Spiroplasma* is endemic to *Gastrimargus marmoratus* (GM).

Non-multidimensional scaling (NMDS) was used to compare the differences between sample groups, and stress less than 0.2 indicated that the NMDS analysis had some reliability. For instance, as shown in Figure 5B, stress = 0.1129, and the result was reliable. The closer the distance of the graphic, the more similar the samples are. Three samples of *Gastrimargus marmoratus* (GM) clustered together and were near *Calliptamus abbreviatus* (CA). Six samples of *Oxya chinensis* (OCb, OCc) collected from the two sites were close to each other and to *Pararcyptera microptera meridionalis* (PM). However, the three samples of *Calliptamus abbreviatus* (CA), *Pararcyptera microptera meridionalis* (PM), and the two grasshopper species were more dispersed. To compare inter-group and inter-individual differences among the four grasshopper species, we performed permutational multivariate analysis of variance (PERMANOVA; Adonis) analysis (Figure 5C). The beta distance of all inter-group samples was greater than the beta distance data of all intra-group samples; that is, the differences in inter-group samples of the four grasshopper species were greater than the differences in intra-group samples, and the differences in gut microbial diversity species were greater than the differences in individuals of the same species (*R*^2^ = 0.479, *F* = 2.38, *p* = 0.003). These results were corroborated by unweighted pair group method with arithmetic mean (UPGMA) analysis.

### Differential analysis between groups

**Figure 6:**
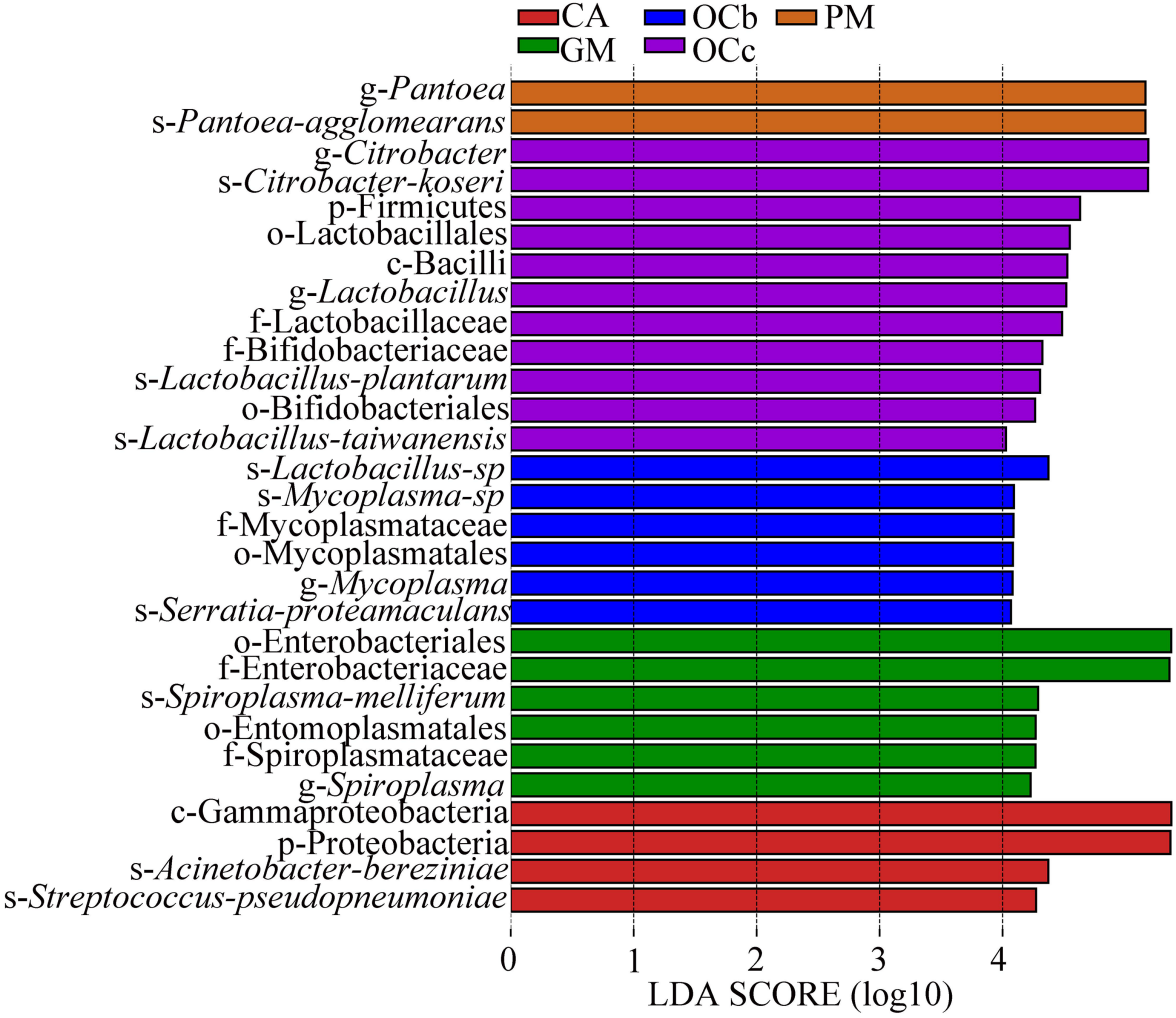
Linear Discriminant Analysis (LDA) score distribution histogram. Bacterial taxa with LDA scores greater than four in the gut microbiota of grasshoppers. *Y*-axis: Features that shown significant difference between groups; *X*-axis: Log10 of LDA score. The features were sorted according to LDA score. A longer bar indicates a more significant difference. The bars were coloured according to the group with highest abundance of corresponding feature. CA, *Calliptamus abbreviatus*; GM, *Gastrimargus marmoratus*; OCc, *Oxya chinensis* in Chengde (c = Chengde); OCb, *Oxya chinensis* in Baoding (b = Baoding); PM, *Pararcyptera microptera meridionalis*.

**Figure 7:**
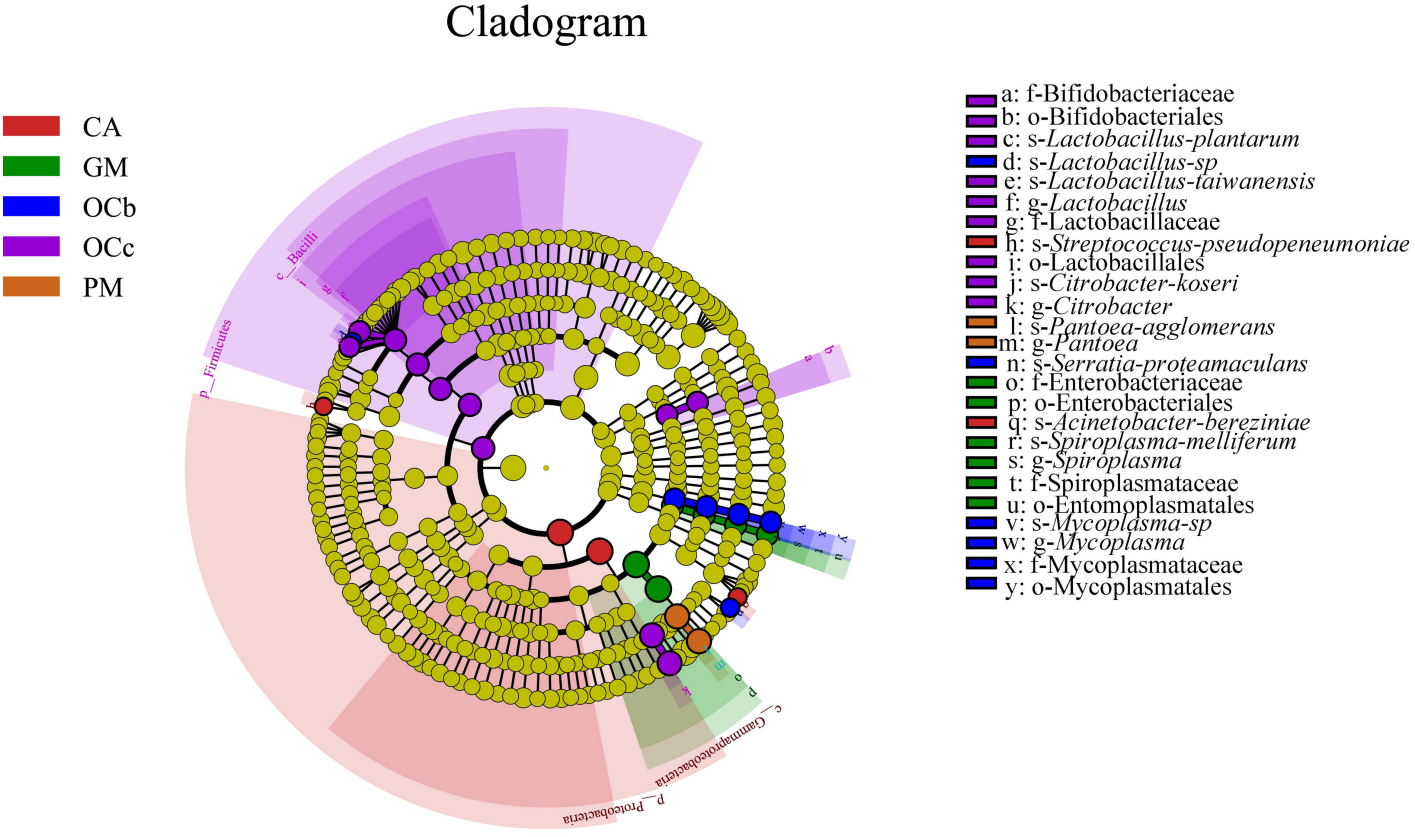
LEfSe analysis cladogram diagram. From the phylum (innermost ring) to species (outermost ring) level, with a linear discriminant analysis (LDA) score > 4. Differential bacterial taxa are marked by lowercase letters. Each small circle at different taxonomic levels represents a taxon at that level, and the diameter of the circle is proportional to the relative abundance. The coloring principle is to color the species with no significant difference as yellow, and the other different species as the group with the highest abundance of the species. CA, *Calliptamus abbreviatus*; GM, *Gastrimargus marmoratus*; OCc, *Oxya chinensis* in Chengde (c = Chengde); OCb, *Oxya chinensis* in Baoding (b = Baoding); PM, *Pararcyptera microptera meridionalis*.

Biomarkers are marker taxa with significant differences in abundance between groups. LDA was used to estimate effective size of each differentially abundant features, i.e., the influence of each feature in the difference. To identify biomarkers with statistical differences between different species of grasshoppers (Segata et al., 2011), linear discriminant analysis (LDA) effect size (LEfSe) was used to screen out different taxa at various levels (kingdom, phylum, class, order, family, genus and species) between different groups based on a standard LDA value greater than four. Finally, bacterial taxa with linear discriminant analysis (LDA) scores greater than four in the gut microbiota of grasshoppers were obtained (Figure 6) (S7 in the Supplementary material). A cladogram from phylum to species was constructed in the meanwhile to fully understand the distribution of these different taxa at various taxonomic levels (Figure 7) (S8 in the Supplementary material). *Oxya chinensis* (OCc) screened the most biomarkers with 11 taxa. *Oxya chinensis* (OCc) screened for biomarkers distributed mainly in Proteobacteria, Firmicutes, Actinobacteria. This was followed by *Oxya chinensis* (OCb), *Calliptamus abbreviatus* (CA) and *Gastrimargus marmoratus* (GM) screened for biomarkers in Proteobacteria, Firmicutes. Only one taxon was screened in *Pararcyptera microptera meridionalis* (PM), and *Pantoea* belonged to the Proteobacteria. The biomarkers screened were the dominant microbiota in the gut of the experimental grasshoppers, and the grasshopper species screened with high numbers of biomarkers were grasshoppers with high gut microbial diversity, which is consistent with previous results. Biomarker screening can provide a basis for future targeted studies of grasshopper gut microbes.

### Functional prediction of gut microbiota

**Figure 8:**
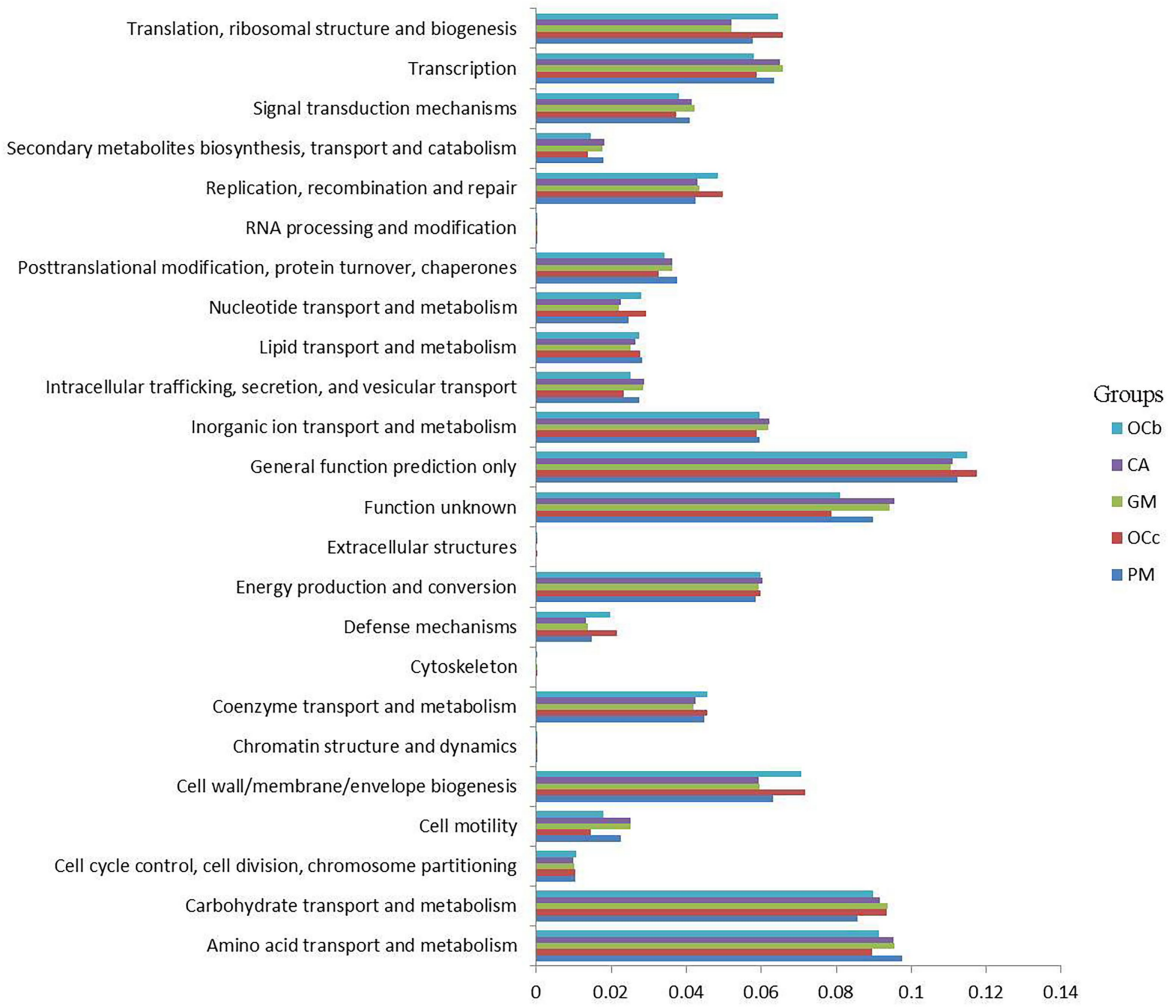
Comparison of predicted the clusters of orthologous groups of proteins (COG) functions of gut bacteria of four species of grasshoppers. CA, *Calliptamus abbreviatus*; GM, *Gastrimargus marmoratus*; OCc, *Oxya chinensis* in Chengde (c = Chengde); OCb, *Oxya chinensis* in Baoding (b = Baoding); PM, *Pararcyptera microptera meridionalis*.

To better understand the important role of grasshopper gut microbiota, we used PICRUSt2 software to predict the functional gene compositions of samples based on 16S rDNA sequencing data and compared them with the cluster of orthologous groups of proteins (COG) database. The results showed that most of the functional prediction categories were related to cellular processes, such as metabolism, replication, and transcription. The major transport and metabolic functions include amino acids, carbohydrates, inorganic ions, energy production and conversion, transcription, replication, recombination, and repair, representing the most active functions in the intestine (Figure 8) (S9 in the Supplementary material). Cellulose degradation is mainly carried out by cellulase produced by anaerobic or aerobic microorganisms during biotransformation (Abudi et al., 2018). In this process, carbohydrate transport and metabolism, inorganic ion transport and metabolism, energy production and transformation, coenzyme transport and metabolism play a dominant role (Artzi et al., 2016).

**Figure 9:**
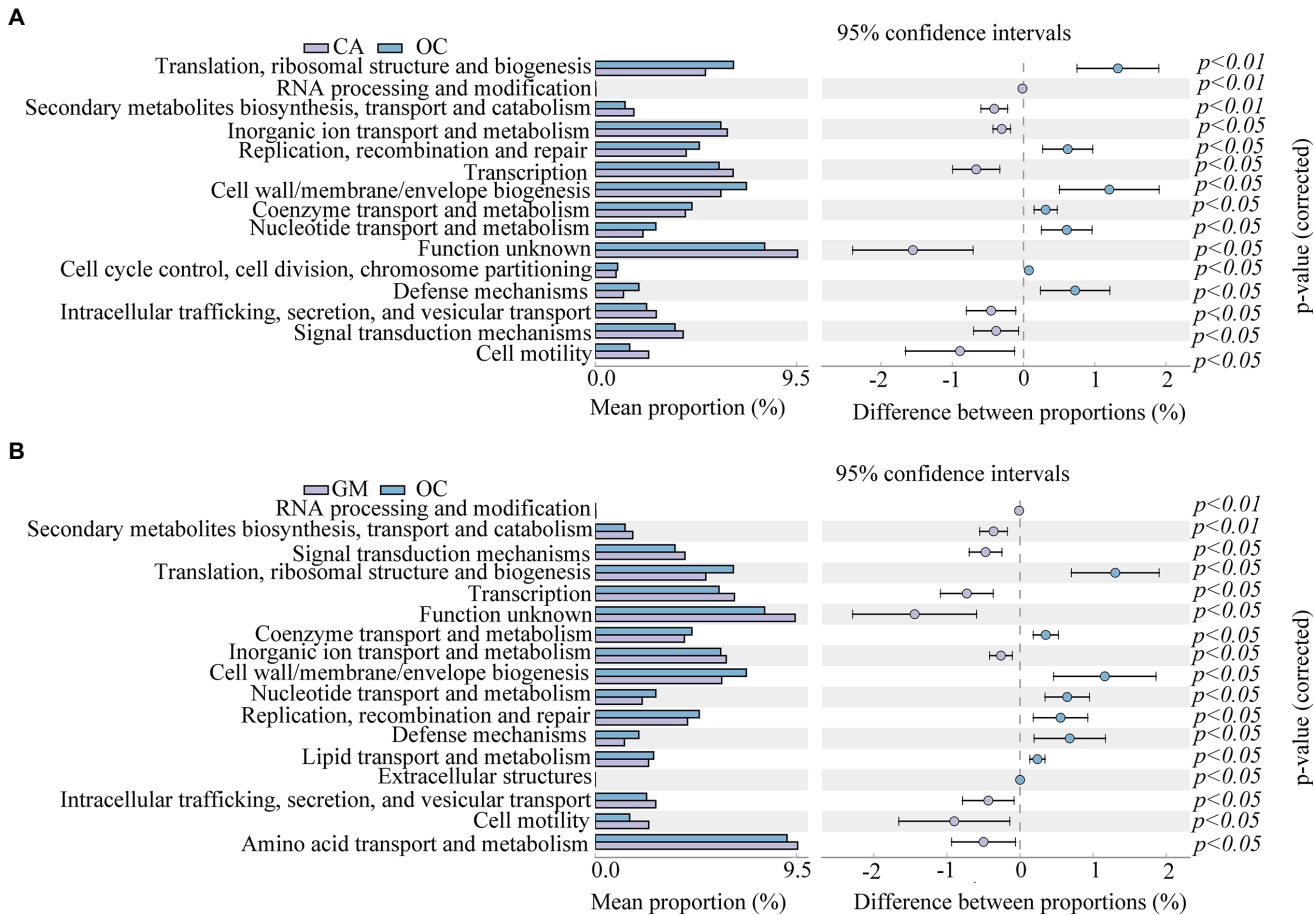
Significant differences (one-way ANOVA, *p* < 0.05.) in functions abundance of **(A)** *Calliptamus abbreviatus* and *Oxya chinensis*, **(B)** *Oxya chinensis* and *Gastrimargus marmoratus.* The left panel shows the abundance ratio of different functions in the two groups of samples, the middle shows the difference ratio of functional abundance within the 95% confidence interval, and the rightmost value is the value of *p*. CA, *Calliptamus abbreviatus*; GM, *Gastrimargus marmoratus*; OC, *Oxya chinensis*.

To better analyze the functional differences between different grasshopper species, a two-by-two test for significant differences was conducted between samples of the four grasshopper species (*α* = 0.05); after testing, *Oxya chinensis* (OCc) and *Oxya chinensis* (OCb) were not significantly different from each other as a function, so the *Oxya chinensis* (OC) samples collected from the two locations were used as a group. Only *Oxya chinensis* (OC) and *Calliptamus abbreviatus* (CA), *Oxya chinensis* (OC), and *Gastrimargus marmoratus* (GM) were significantly functionally different from each other, and the remaining three combinations of two and two grasshoppers were not significantly different from each other. *Oxya chinensis* (OC) was significantly different from *Calliptamus abbreviatus* (CA) in secondary metabolite biosynthesis, transport and catabolism, translation, ribosome structure, and biogenesis, RNA processing and modification (Figure 9A; *p* < 0.01). *Oxya chinensis* (OC) was significantly different from *Gastrimargus marmoratus* (GM) in RNA processing and modification, secondary metabolite biosynthesis, transport and catabolism (Figure 9B; *p* < 0.01). Additionally, *Oxya chinensis* (OC) was significantly different than *Calliptamus abbreviatus* (CA) and *Gastrimargus marmoratus* (GM) with respect to cell motility, cell wall/membrane/envelope biogenesis; coenzyme transport and metabolism; defense mechanisms; inorganic ion transport and metabolism; intracellular trafficking, secretion, and vesicular transport; nucleotide transport and metabolism; replication, recombination and repair; signal transduction mechanisms; transcription (Figures 9A,B; *p* < 0.05). In cell cycle control, cell division, chromosome partitioning (Figure 9A; *p* < 0.05), there were significant differences between *Oxya chinensis* (OC) and *Calliptamus abbreviatus* (CA). In translation, ribosomal structure and biogenesis, lipid transport and metabolism, extracellular structures, amino acid transport and metabolism (Figure 9B; *p* < 0.05), there were significant differences between *Oxya chinensis* (OC) and *Gastrimargus marmoratus* (GM).

### Digestibility of cellulose and hemicellulose

The cellulose content of wheat seedlings was 63.87% and the hemicellulose content was 9.48%. The digestibility of cellulose and hemicellulose in the grasshopper locusts is shown in Table 5. The digestibility of cellulose by the four grasshopper species was higher than that of hemicellulose. In descending order, cellulose digestibility was *Pararcyptera microptera meridionalis* (PM), *Gastrimargus marmoratus* (GM), *Oxya chinensis* (OCb), *Calliptamus abbreviatus* (CA), and *Oxya chinensis* (OCc). Hemicellulose digestibility was from *Calliptamus abbreviatus* (CA), *Oxya chinensis* (OCc), *Gastrimargus marmoratus* (GM), *Oxya chinensis* (OCb), and *Pararcyptera microptera meridionalis* (PM) in descending order. The cellulose digestibility of *Oxya chinensis* collected from different geographical areas did not differ significantly, but the hemicellulose digestibility differed significantly, which may be related to the living environment. The significant differences (*p* < 0.01) in cellulose and hemicellulose digestibility among the various grasshoppers may be due to differences in the species and number of microorganisms that break down cellulose and hemicellulose intestinally.

**Table 5:** Cellulose and hemicellulose digestibility by grasshopper species.

Species	Abbreviation	Cellulose digestibility (%)	Hemicellulose digestibility (%)
*Oxya chinensis* (Thunberg, 1815)	OCc	51.16 ± 0.02	32.41 ± 0.03
	OCb	45.93 ± 0.06	7.66 ± 0.02
*Pararcyptera microptera meridionalis* (Ikonnikov, 1911)	PM	65.21 ± 0.04	7.56 ± 0.03
*Gastrimargus marmoratus* (Thunberg, 1815)	GM	62.30 ± 0.04	29.76 ± 0.04
*Calliptamus abbreviatus* Ikonnikov, 1913	CA	47.33 ± 0.08	44.44 ± 0.14

OCb and OCc are abbreviations of *Oxya chinensis* collected in Baoding, Hebei and Chengde, Hebei, respectively, b = Baoding, c = Chengde.

### Correlation analysis of intestinal microorganism of grasshoppers with digestibility of cellulose and hemicellulose

**Figure 10:**
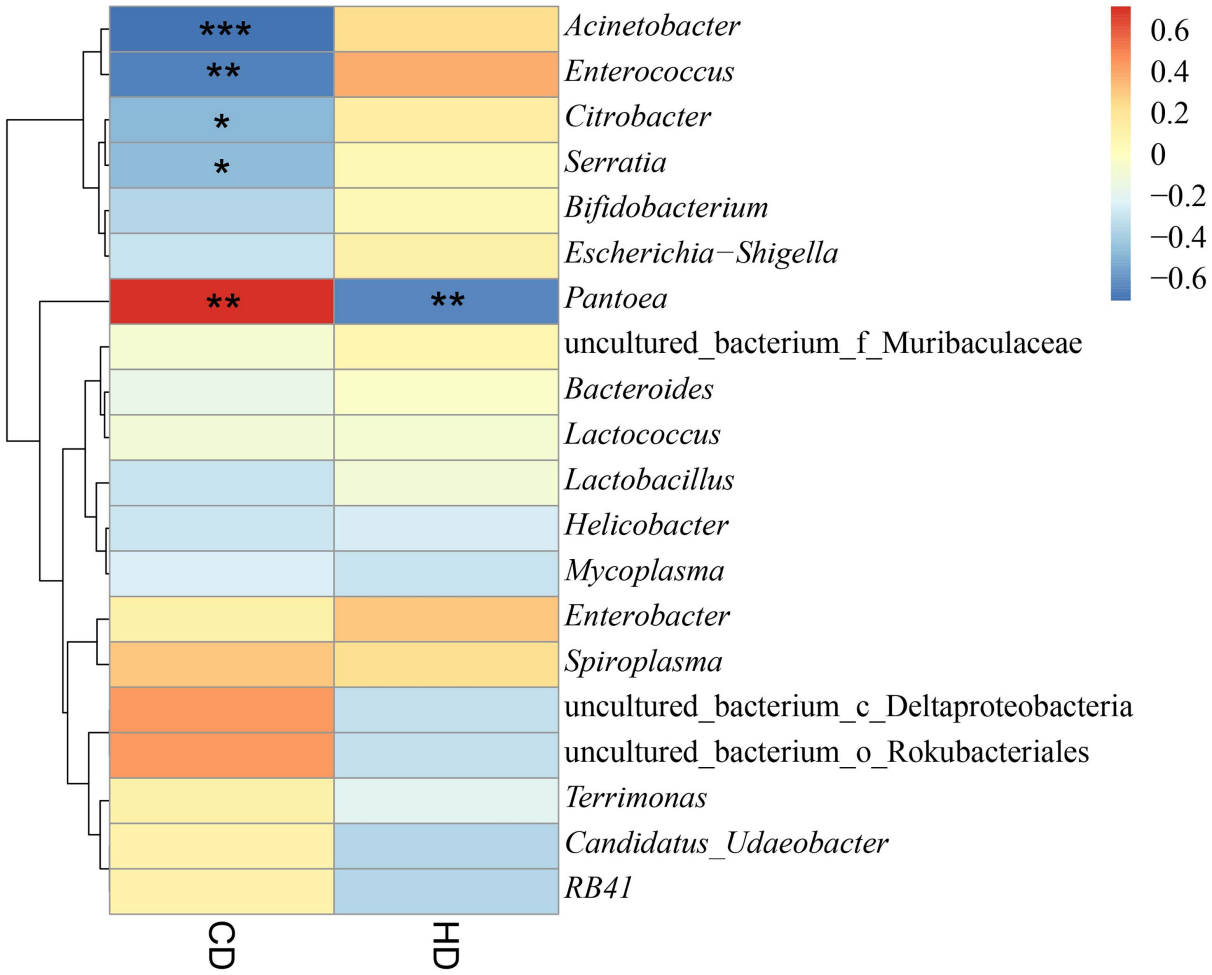
Heatmap of the correlation between digestibility and bacterial abundance dendrograms. CD, cellulose digestibility; HD, hemicellulose digestibility. A number of “*” above 2 is highly significant correlation. A “*” indicated significant correlation.

The relationship between grasshopper gut microbes and cellulose digestion was investigated by conducting Spearman correlation analysis between cellulose and hemicellulose digestibility and the gut microbes of grasshoppers (S10 and S11 in the Supplementary material). As shown in Figure 10, there was a significant positive correlation with cellulose digestibility for *Pantoea*. A significant negative correlation was found with cellulose digestibility for *Acinetobacter*, *Enterococcus*, *Citrobacter*, *Serratia*. A significant negative correlation was found with hemicellulose digestibility for *Pantoea*, and a positive correlation with hemicellulose digestibility for *Enterococcus*, *Enterobacter*, and *Acinetobacter*. The results showed that four grasshopper gut microorganisms were correlated with cellulose and hemicellulose digestibility, and *Pantoea*, *Acinetobacter*, *Enterobacter*, *Serratia*, and *Citrobacter* were the dominant genera and may be involved in cellulose and hemicellulose metabolic activities. In the future, the genus of bacteria related to cellulose and hemicellulose degradation in the gut of grasshoppers can be the focus of study, laying the foundation for developing the potential value of grasshoppers as a bioreactor for cellulose decomposition. This will also provide new ideas for the decomposition and utilization of agricultural straw, and influence the digestion and metabolism of grasshoppers for control purposes.

## Discussion

The results of this study showed that *Oxya chinensis* had the highest microbiota diversity among the four grasshopper species, and the least diverse was *Gastrimargus marmoratus* microbiota; this result is consistent with the results of Liu (2012). Insect gut microorganisms are influenced by feeding habits, taxonomic status, gut composition, physiological structure, and other factors (Hammer et al., 2017; Smith et al., 2017; Zheng et al., 2021), and their species composition and proportions change dynamically and therefore vary between individuals and regions. Tsagmo Ngoune et al. (2019) found that the gut microbial communities of *Glossina palpalis palpalis* did not differ significantly between the two regions, and that their core dominant microbiota did not change. In the present study, there were no significant differences in the gut microbiota of *Oxya chinensis* from the same species collected from different regions, but they had endemic bacteria under the influence of various factors, which demonstrated the stability of the insect gut microbiota among the same species.

In this study, the interspecific differences in the gut microorganisms of the four grasshoppers were greater than the intraspecific differences, and the dominant phyla were Proteobacteria, Bacteroidetes, and Firmicutes. The dominant genus among the different species of grasshoppers was *Enterobacter*, but *Citrobacter*, *Bacteroides*, *Pantoea*, and *Helicobacter* were also present genera in the gut of the four species of grasshoppers. Proteobacteria is one of the most dominant groups of gut bacteria (Yun et al., 2014) despite the differences in the dominant phyla of insect gut bacteria among different species. Wang et al. (2019) studied the gut microbial diversity of *Nilaparvata lugens* and found that the bacteria populations were dominated by Proteobacteria, Bacteroidetes, and Firmicutes. Additionally, Dong et al. (2021) found that the dominant phylum in the gut of *Apis cerana* is Proteobacteria. In addition, Suárez-Moo et al. (2020) found that the dominant bacteria in *Copris incertu*s were *Enterobacter* and *Serratia*. Among the gut microorganisms of various species of termites, Firmicutes, Actinomycetes, and Bacteroidetes are the dominant groups (Li et al., 2017). Analysis of the dominant microbiota of grasshoppers in this study also supports these results.

Muratore et al. (2020) studied the gut bacterial communities of six species of grasshoppers in coastal tallgrass prairies and found that Actinobacteria, Proteobacteria, and Firmicutes were the core bacteria of their gut and were conserved. Wang et al. (2020) studied the intestinal microbial diversity of three species of grasshoppers—*Oedaleus decorus asiaticus*, *Aiolopus tamulus*, and *Shirakiacris shirakii*—and found that the dominant phyla of the three species of grasshoppers were Proteobacteria and Firmicutes, and the dominant genera were *Klebsiella*, *Enterobacter*, *Enterococcus*, and *Pantoea*. Most of the intestinal microbiota in *Schistocerca gregaria* belonged to Proteobacteria, and the diversity of Proteobacteria had a positive effect on improving the defense ability of *Schistocerca gregaria* (Dillon et al., 2010). Mead et al. (1988) studied the intestinal microorganisms of *Melanoplus sanguinipes* and found that there were four main groups of intestinal microorganisms: *Enterococcus* of Firmicutes, *Pseudomonas*, *Serratia* and *Enterobacter* of Proteobacteria, the largest number of *Enterococcus* belonging to Firmicutes. Zhao et al. (2008) studied the intestinal bacteria of the fifth nymph of *Locusta migratoria* reared indoors, with *Klebsiella* and *Enterobacter cloacae* as the dominant resident microbiota. In contrast, the dominant intestinal flora of *Schistocerca gregaria*, which feeds mainly on plant leaves or stalks, is *Enterobacter* and *Streptococcus* (Dillon and Charnley, 2002). There were significant differences in the dominant intestinal microbiota among the grasshopper species, which corroborates the results of this research.

The results of this study showed that the cellulose digestibility of the four grasshopper species was higher than the hemicellulose digestibility, which was consistent with the results of Zhao (2015). Determination of cellulose and hemicellulose digestibility of corn straw in *Locusta migratoria*, and the decomposition rates of cellulose and hemicellulose in corn straw by adult *Locusta migratoria* were 15.1 and 3.90%, respectively. Wang et al. (2020) determined the cellulose digestibility of three species of grasshoppers, *Aiolopus tamulus*, *Oedaleus decorus asiaticus*, and *Shirakiacris shirakii*, to wheat at 43.95, 38.01, and 44.12%, respectively, and the hemicellulose digestibility of wheat at 17.21, 24.99, and 47.65%, respectively. The digestibility of four grasshopper species, *Acrida cinerea*, *Trilophidia annulata*, *Atractomorpha sinensis*, and *Sphingonotus mongolicus*, ranged from 49.87 to 67.91% for wheat, and hemicellulose digestibility ranged from 17.77 to 47.51% (Bai et al., 2022). The cellulose digestibility of wheat by the four grasshopper species measured in this research ranged from 45.93 to 65.21%, which was higher than the cellulose digestibility of the above grasshoppers; the hemicellulose digestibility ranged from 7.56 to 32.41%, which was lower than the above grasshoppers. Tian and Ba (2017) found that the rumen fluid of Tibetan sheep had a 25.8% digestibility of cellulose from *Hordeum vulgare*. Li et al. (2000) studied the digestibility of sheep to different treatments of corn straw and found that the digestibility of crude fiber ranged from 34.21 to 61.21%. Moreover, Zhang et al. (2019) found that Holstein cows digested 28.5–30.9% of neutral detergent fiber and 29.1–37.0% acid detergent fiber from wheat straw. In addition, the addition of cellulase increases the digestibility of cellulose in roughage (corn straw and wheat bran) in Hu sheep and Holstein cows, ranging from 59.15–67.29% and 47.43–64.54%, respectively (Xiao et al., 2020). The cellulose digestibility measured in this study was comparable or higher than that in mammals. The digestion and utilization of cellulose and hemicellulose in the four grasshopper species were high. This result may be related to the feeding material, experimental methods, and the intestinal microorganisms.

Gut microorganisms of insects and ruminants play an important role in the degradation of lignocellulose, and synergistic microbial symbioses can make a significant contribution to the recovery of renewable carbon from lignocellulose, which is difficult to degrade (Rajeswari et al., 2021). *Enterobacter*, *Bacillus* and *Klebsiella* isolated from *Spodoptera frugiperda*, *Proisotoma ananevae*, and *Helicoverpa armigera* have cellulose-degrading abilities (Wang et al., 2018; Li et al., 2020; Dar et al., 2021). *Bacillus flexus* has been shown to be a potential cellulose-degrading bacterium with additional lignocellulose-degrading ability (Sanchez-Gonzalez et al., 2011; Sreeremya and Rajiv, 2017; Kumar et al., 2020). Nelson et al. (2020) isolated a cellulose-decomposing *Bacillus* species from the intestinal tract of *Schistocerca gregaria*, which proved that the *Bacillus* did have a gene encoding cellulase. Moreover, *Klebsiella* has been isolated from the hindgut of crickets and has cellulolytic capacity (Zhang et al., 2020). *Enterobacter asburiae* can efficiently produce ethanol from hemicellulose hydrolysate (Bi et al., 2009). Notably, *Bacteroides thetaiotaomicron*, which has a large proportion and relative abundance in the intestine of *Oxya chinensis*, plays a role in cellulose degradation (Zhang and Wang, 2009), which provides a basis for future studies on cellulose-degrading bacteria. In this study, the Spearman correlation analysis was performed between cellulose digestibility, hemicellulose digestibility, and intestinal microorganisms of four grasshopper species, which also proved that intestinal microorganisms are related to cellulose degradation, and that locusts have the potential to be developed as bioreactors for cellulose decomposition, providing new ideas for the decomposition and utilization of straw in agriculture and animal husbandry.

In conclusion, the gut microbial diversity of four grasshopper species, *Oxya chinensis*, *Pararcyptera microptera meridionalis*, *Calliptamus abbreviatus*, and *Gastrimargus marmoratus*, were analyzed and functionally predicted. Collectively the four grasshopper species had the following three dominant phyla: Proteobacteria, Bacteroidetes, and Firmicutes, and the common dominant genus was *Enterobacter*. There were no significant differences in gut microbial diversity between the two geographic collections of *Oxya chinensis*. It was also demonstrated that there was a correlation between gut microbe composition and cellulose and hemicellulose digestibility. In the future, we will focus on the utilization of functional genes in grasshopper gut microbes and the study of bacterial genera related to cellulose and hemicellulose degradation in the gut.

## Data availability statement

The datasets presented in this study can be found in online repositories. The names of the repository/repositories and accession number(s) can be found at: NCBI: PRJNA863334.

## Author contributions

YL conceived and designed the experiments, performed the experiments, analyzed the data, authored drafts of the paper, and approved the final draft. W-JL and F-FL analyzed the data, reviewed drafts of the paper, and approved the final draft. X-BX and Y-YG analyzed the data, prepared figures, and approved the final draft. LW and KL prepared tables and approved the final draft. X-JL conceived and designed the experiments, acquired funding, reviewed drafts of the paper, and approved the final draft. All authors contributed to the article and approved the submitted version.

## Funding

This research was funded by the National Natural Science Foundation of China (Nos. 32070473 and 31872274) and the Hebei Provincial Innovation Capacity Enhancement Program-Special Project for High-level Talent Team Building (No. 225A2904D).

## Conflict of interest

The authors declare that the research was conducted in the absence of any commercial or financial relationships that could be construed as a potential conflict of interest.

## Publisher’s note

All claims expressed in this article are solely those of the authors and do not necessarily represent those of their affiliated organizations, or those of the publisher, the editors and the reviewers. Any product that may be evaluated in this article, or claim that may be made by its manufacturer, is not guaranteed or endorsed by the publisher.
